# Assessment of the Influence of Acetic Acid Residue on Type I Collagen during Isolation and Characterization

**DOI:** 10.3390/ma11122518

**Published:** 2018-12-11

**Authors:** Seon Young Bak, Sang Woo Lee, Chong Hyuk Choi, Hyun Woo Kim

**Affiliations:** 1 Graduate Program of Nano Science and Technology, Graduate School of Yonsei University, Seoul 03722, Korea; psyighmn@gmail.com; 2 Center for Biomaterials, Biomedical Research Institute, Korea Institute of Science and Technology, Seoul 02792, Korea; 3 Biomedical Engineering Research Center, Asan Institute for Life Sciences, Asan Medical Center, Seoul 05505, Korea; upps1978@gmail.com; 4 Department of Orthopaedic Surgery, Yonsei University College of Medicine, Seoul 03722, Korea

**Keywords:** type I collagen, isolation, dialysis, acetic acid, porous scaffold, adipose-derived stem cells (ADSCs)

## Abstract

Various methods for isolation of type I collagen using acids, bases, enzymes, and their combinations have been applied. However, a lack of standardization exists among type I collagens isolated by various approaches. Consequently, in this study, we assessed the influence of acetic acid residue on type I collagen isolated by pepsin-acetic acid treatment, the fabrication of collagen-based porous scaffolds, and the seeded cells on collagen scaffolds. Unlike the isolated collagen dialyzed by deionized water (DDW), collagen dialyzed by 0.5 M acetic acid (DAC) exhibited structural and thermal denaturation. Both DDW- and DAC-based porous scaffolds at all collagen concentrations (0.5, 1 and 2% *w*/*v*) showed the high degree of porosity (>98%), and their pore morphologies were comparable at the same concentrations. However, the DDW- and DAC-based collagen scaffolds displayed significant differences in their physical properties (weight, thickness, and volume) and swelling behaviors. In particular, the weight losses induced by mechanical stimulation reflected the high degradation of DAC-collagen scaffolds. In cell culture experiments using adipose-derived stem cells (ADSCs), the characteristics of mesenchymal stem cell (MSC) did not change in both DDW- and DAC-collagen scaffolds for 10 days, although cells proliferated less in the DAC-collagen scaffolds. Our results suggest that the elimination of acetic acid residue from isolated collagen is recommended to produce collagen scaffolds that provide a stable environment for cells and cell therapy-related applications.

## 1. Introduction

Type I collagen is considered one of the most useful biomaterials and is used extensively in the field of tissue engineering [1,2,3,4]. Accordingly, its isolation at both laboratory and commercial scales is important [5,6] and various methods using acids, bases, enzymes, and their combinations have been applied to isolate type I collagen [7]. Among various methods, the treatment with pepsin-acetic acid solution is the most widely used approach and highly reliable for medical purposes [8,9,10]; this treatment retains the triple-helical domain of collagen, but lowers its immunogenicity by cleaving its telopeptides [9,11]. 

However, a lack of standardization exists among type I collagens isolated by various approaches [6]. Specifically, the variability is introduced in isolated collagens due to alterations in the structural and thermal properties of collagen induced by acetic acid [12,13] and due to variation in the concentrations of acetic acid used in the isolation process. Moreover, the resulting influences on the characteristics of the collagen-based scaffolds and the seeded cells cultured on the scaffolds have been not proved clearly. Despite the considerable numbers of studies that support the use of collagen as the primary material for the fabrication of scaffolds, the routine use of isolated collagen without prior characterization is controversial.

In this study, we aimed to determine the influence of acetic acid residue on the characteristics of isolated collagen. Moreover, to elucidate the influence of acetic acid residue on collagen-based porous scaffolds and the culturing cells on the scaffolds, we intended to demonstrate the relationships between the material used for scaffolds and the seeded cells.

For this purpose, we isolated type I collagen from porcine skin by pepsin-acetic acid treatment and dialyzed it against deionized water (DDW) and 0.5 M acetic acid (DAC). The structural and thermal properties of both DDW- and DAC-collagens were confirmed by comparison to those of commercially available type I collagen. Three different concentrations of isolated collagen were used to fabricate collagen-based porous scaffolds, and their morphological and physical characteristics as well as swelling and degradation behaviors were analyzed. Adipose-derived stem cells (ADSCs) were seeded on the fabricated scaffolds, and the relationships between the collagen scaffolds and the seeded cells were evaluated by analyzing the genetic maintenance and proliferation of the seeded cells.

## 2. Materials and Methods

### 2.1. Materials

Porcine skin, used in food, was purchased from an open market (Seoul, Korea). It was chosen as the raw material for type I collagen isolation due to its high similarity to human skin [14,15]. Glacial acetic acid was purchased from Duksan (Seoul, Korea), and ethanol, pepsin (EC 3.4.23.1, 1015 units/mg protein) from porcine stomach mucosa, 1-ethyl-3-(3-dimethylaminopropyl) carbodiimide (EDC), ninhydrin solution, and sodium chloride (NaCl) were purchased from Sigma-Aldrich (St. Louis, MO, USA). All other chemicals used were of analytical grade, and water was deionized before use.

### 2.2. Isolation and Characterization of Type I Collagen

#### 2.2.1. Isolation of Type I Collagen from Porcine Skin

Type I collagen was isolated by acetic acid-pepsin treatment, as described previously [16]. The isolation procedure was carried out at 4 °C. Porcine skin (10 g) was homogenized in 1 L of 0.5 M acetic acid, and 2 g of porcine pepsin was then added and stirred for 24 h. After centrifugation (all centrifugation was performed at 10,700× *g* for 10 min), the supernatant was salted out by 640 mL of 5 M NaCl solution. The precipitate was suspended in 100 mL of ethanol with stirring for 24 h, after which it was recovered by centrifugation and then re-suspended in 50 mL of ethanol. After centrifugation, the precipitate was suspended in 50 mL of 0.5 M acetic acid and then transferred to a dialysis tubing cellulose membrane (molecular weight cut-off: 14 kDa; Sigma-Aldrich) and dialyzed against 900 mL of 0.5 M acetic acid for 3 days by changing the solution every 12 h. The molar concentration of acetic acid in the surrounding solution was measured by titration with 0.1 M sodium hydroxide solution. When the molar concentration of acetic acid in the surrounding solution was constantly sustained at 0.5 M for 72 h, the final collagen was referred to as DAC. Collagen that was dialyzed against deionized water (DW) was referred to as DDW when the molar concentration of acetic acid in the surrounding solution was less than 0.0001 M at 72 h. The resulting collagen solutions were frozen at −20 °C and then lyophilized.

#### 2.2.2. Gel Electrophoresis

Sodium dodecyl sulfate-polyacrylamide gel electrophoresis (SDS-PAGE) was performed using the discontinuous Tris-HCl/glycine buffer system, according to the method of Laemmli [17]. The protein patterns of the collagens isolated from porcine skin were compared against a commercially available ultrapure type I collagen (PC-001, Dalim Tissen, Seoul, Korea). Briefly, the collagen samples were electrophoresed using an 8% separating gel and a 5% stacking gel at a constant voltage of 120 V and a maximum current of 400 mA for 1.5 h. The gel was then stained using 0.1% *w*/*v* Coomassie brilliant blue R-250, containing 50% *v*/*v* methanol and 10% *v*/*v* acetic acid, and destained with 40% *v*/*v* methanol and 10% *v*/*v* acetic acid. A high molecular weight protein marker (ThermoFisher Scientific, Waltham, MA, USA) was used to estimate the molecular weight of the proteins.

#### 2.2.3. Raman Spectroscopy

Raman spectroscopy (LabRam ARAMIS, Horiba Jobin Yvon, Edison, NJ, USA) was carried out using a 532-nm laser at a power of 50 mW with an exposure time of 10 s. The Raman spectra were analyzed after normalization of peak intensities to the amide I band at around 1660 cm^−1^.

#### 2.2.4. X-ray Diffraction (XRD) Analysis

The spacing of the collagen fibers and phase identification of the two isolated collagens (DDW- and DAC-collagen) and the commercial collagen were determined using thin-film XRD. To fabricate the thin films, 0.5 mL of 1% *w*/*v* collagen solution was poured over a cover glass (18 × 18 mm^2^) and placed in a refrigerator at 4 °C to dry. The high-resolution XRD system (Rigaku, Tokyo, Japan) was operated at 45 kV and 200 mA using CuKα radiation (λ = 0.15418 nm).

#### 2.2.5. Simultaneous Thermal Analysis (STA)

The thermodynamic properties of DDW-, DAC-, and the commercial collagen pellets (23 mg, 8 mm diameter) in the dehydrated state were evaluated by thermal gravimetric analysis (TGA) and differential scanning calorimetry (DSC), performed using a simultaneous thermal analyzer (STA8000, PerkinElmer, Waltham, MA, USA). The sample was heated from 20 to 500 °C at 1 °C/min in N_2_ gas.

### 2.3. Preparation and Characterization of Porous Scaffolds

#### 2.3.1. Preparation of Porous Scaffolds

Collagen solutions (0.5, 1 and 2% *w*/*v*) were prepared from DDW and DAC and then homogenized in 50-mL conical tubes by vortexing for 5 min at 4 °C. Air bubbles were removed from the solutions by centrifugation at 3000× g for 3 min. Finally, 380 μL of each collagen solution was placed into one well of a 24-well plate, frozen at −20 °C, and lyophilized for 1 day each. For cross-linking, the fabricated porous scaffolds were immersed in 50 mM EDC solution in 95% *v*/*v* ethanol at room temperature for 24 h [18]. The cross-linked scaffolds were then withdrawn and sonicated in 50 mL of DW for 20 s three times to remove residual EDC. The washed scaffolds were re-frozen at −20 °C and then lyophilized for 1 day each.

#### 2.3.2. Morphology

The surface morphologies of the porous scaffolds were characterized by a field emission scanning electron microscope (FE-SEM; JEOL-7001F, JEOL Ltd., Tokyo, Japan). Each sample was sputter-coated with Pt and visualized by SEM at 15 kV. The pore size was measured from the SEM images by ImageJ software (National Institutes of Health (NIH), Bethesda, MD, USA), and 40 pores were assessed.

#### 2.3.3. Physical Characteristics

The weights of the scaffolds were measured by an electronic balance (Mettler-Toledo, Greifensee, Switzerland) with 0.1 mg readability. The porosity and volume of the final products were measured by a displacement method with ethanol [19]. The thickness was calculated after measuring the diameter using a stainless-steel ruler with 0.5-mm increments. For all parameters, three individual scaffolds were analyzed.

#### 2.3.4. Free Amine Index

The free amine indices of the cross-linked scaffolds were determined using ninhydrin solution [20]. Approximately 7–13 mg of each sample was immersed in 100 μL of 0.05% *v*/*v* acetic acid, and then 300 μL of DW and 100 μL of ninhydrin solution were added sequentially. The solutions were mixed and placed in a boiling water bath for 10 min. After cooling to room temperature, 100 μL of 95% *v*/*v* ethanol was added, and the solutions were re-mixed. A blank was established using DW instead of the sample solution. The absorbance of the solutions was measured at 570 nm using a spectrophotometer (VERSAmax tunable microplate reader, Molecular Devices, Sunnyvale, CA, USA), and the data were analyzed using SoftMax pro (version 6.2.2, Molecular Devices). The free amine index was calculated by the following equation:

(1) $$\mathrm{Free}\;\mathrm{amine}\;\mathrm{index}\;=\frac{A570}{\mathrm{Wd}}$$

where A570 is the absorbance at 570 nm and Wd is the weight of the dry scaffold.

#### 2.3.5. Swelling Test

The water absorption (swelling ratio) of the scaffolds was analyzed by immersing the dry scaffolds in DW at room temperature for 1 day. The weights of the swollen scaffolds were measured after removing the excess surface solution with filter paper. The ratio of water absorption was calculated by the following equation:

(2) $$\mathrm{Water}\;\mathrm{absorption}\;\left(\%\right)\;=\frac{\mathrm{Ws}-\mathrm{Wd}}{\mathrm{Wd}}\times 100$$

where Wd is the weight of the dry scaffold and Ws is the weight of the swollen scaffold.

#### 2.3.6. In Vitro Degradation

The porous scaffolds were placed into individual wells of a 12-well plate containing 1.5 mL 1× PBS (pH 7.4) and incubated at 37 °C with orbital shaking at 120 rpm achieved by a Heidolph Rotamax 120 orbital shaker (Wolf Labs, York, UK). After 1, 4, 7 and 14 days of incubation, samples were removed from the well, rinsed five times with DW, and then lyophilized and weighed. The percentage of weight loss was calculated by the following equation:

(3) $$\mathrm{Weight}\;\mathrm{loss}\;\left(\%\right)\;=\;\frac{\mathrm{Wi}-\mathrm{Wf}}{\mathrm{Wi}}\times 100$$

where Wi and Wf are the initial and final weights of the dry scaffold, respectively.

### 2.4. Cell Culture Studies

#### 2.4.1. Isolation and Cultivation of ADSCs

Human ADSCs were obtained from the Department of Surgery, Yonsei University College of Medicine. The human tissue harvest protocols were approved by the Institutional Review Board (IRB, 4-2010-0236) of Severance Hospital. The ADSCs were incubated at 37 °C in 5% CO_2_ in conditioned medium consisting of 60% Dulbecco’s Modified Eagle Medium-low glucose (DMEM-LG, GibcoBRL, Grand Island, NY, USA), 40% MCDB-201 medium (Sigma-Aldrich, St. Louis, MO, USA), 10^−4^ M ascorbic acid 2-phosphate (Sigma-Aldrich, St. Louis, MO, USA), and 1% antibiotic/antimycotic solution (GibcoBRL, Crewe, UK) with 10% fetal bovine serum (FBS, WelGENE, Daegu, Korea).

#### 2.4.2. Cell Seeding on Porous Scaffolds

Each of the cross-linked scaffolds was sterilized with 70% *v*/*v* ethanol for 30 min and 40-W ultraviolet light for 20 min. Each scaffold was then washed with DW and 1× PBS (pH 7.4) and subsequently incubated in 1× PBS (pH 7.4) for 1 day. Before cell seeding, the scaffolds were rinsed with conditioned medium consisting of 60% DMEM-LG (GibcoBRL), 40% MCDB-201 medium (Sigma-Aldrich, St. Louis, MO, USA), 10^−4^ M ascorbic acid 2-phosphate (Sigma-Aldrich, St. Louis, MO, USA), and 1% antibiotic/antimycotic solution (GibcoBRL) with 10% fetal bovine serum (FBS, WelGENE, Daegu, Korea). For optical confirmation, ADSCs were stained with 4′,6-diamidino-2-phenylindole (DAPI, Invitrogen, Carlsbad, CA, USA), seeded on scaffolds in 24-well culture plates at a final concentration of 1.0 × 10^5^ cells, and allowed to attach for 1 day. Then, the ADSC-seeded scaffolds were transferred to another 24-well culture plate.

#### 2.4.3. Cell Proliferation Study

For cell counting, 1.0 × 10^5^ ADSCs were seeded on DDW- and DAC-collagen scaffolds (6 mm diameter, 2 mm thickness). The cell-seeded scaffolds were then transferred into individual wells of a 96-well plate. Cell Counting Kit-8 (CCK-8) solution was added to each well, and the plate was then incubated for 3 h in a humidified incubator (37 °C, 5% CO_2_). Cell proliferation capacity was measured by absorbance at 450 nm using a spectrophotometer (VERSAmax tunable microplate reader, Madison, NC, USA). The proliferation assay was performed at 1, 4, 7 and 14 days of cultivation per passage until two sub-cultivations.

#### 2.4.4. Reverse Transcriptase-Polymerase Chain Reaction (RT-PCR) Analysis

RT-PCR was performed at 10 days after seeding using RNA extracted from cells by the Trizol (Invitrogen, Seoul, Korea) isolation method. The primer sequences and conditions are listed in Table 1.

### 2.5. Statistical Analysis

All quantitative data of porous scaffolds were presented as mean ± SD of three independent experiments. Statistical analysis was performed using two-way ANOVA followed by Bonferroni post-hoc test using GraphPad Prism 5 (GraphPad Software Inc., San Diego, CA, USA). The level of significance for all statistical analyses was set at *p* < 0.05.

## 3. Results

### 3.1. Isolated Type I Collagens

#### 3.1.1. Gel Electrophoresis Patterns of Collagens

**Figure 1:**
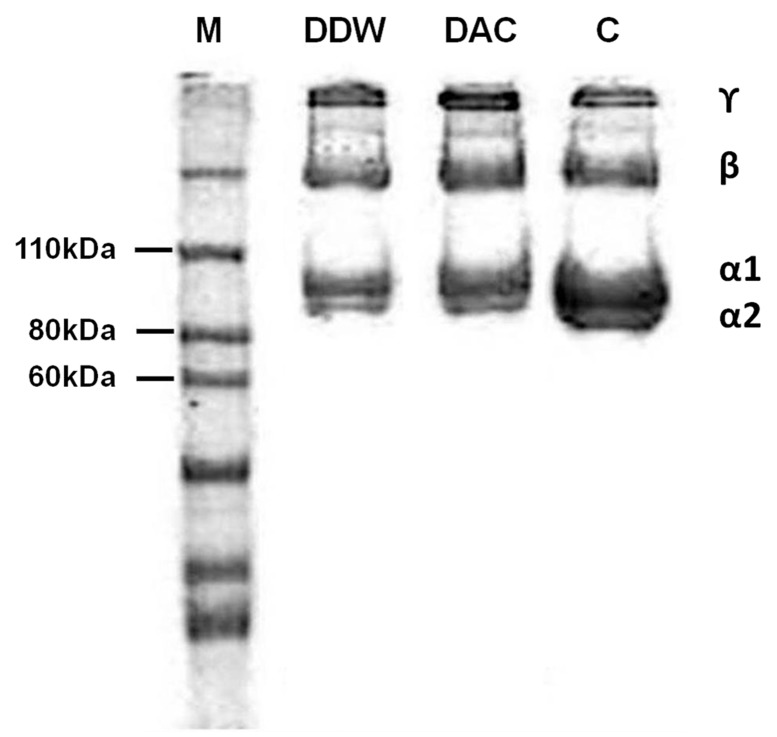
Protein patterns of collagens. SDS-PAGE bands were visualized using Coomassie brilliant blue staining. M, marker.

Gel electrophoretic analysis (Figure 1) showed that both two isolated collagens exhibited the typical protein patterns for type I collagen [21]. Proportions of the β and γ chains for both DDW- and DAC-collagen were relatively higher than for the commercial collagen (C in Figure 1), while the proportions of α1, α2, β, and γ chains in DDW- and DAC-collagen were comparable.

#### 3.1.2. Raman Spectra of Collagens

**Figure 2:**
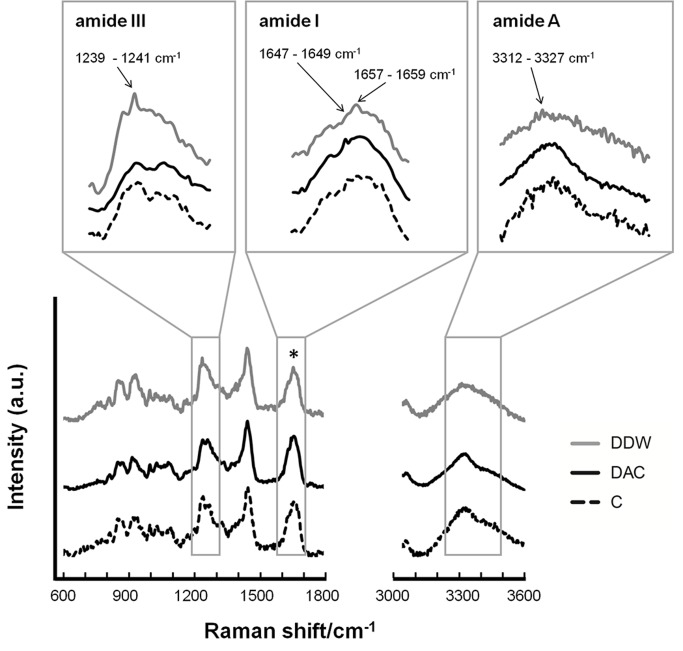
Structural characteristics of collagens. Raman spectra of DDW-, DAC- and commercial collagen (C) were normalized to the amide I band at around 1660 cm^−1^ (the peak marked as *).

Results for the structural and conformational characteristics of the collagens were obtained by Raman spectroscopy (Figure 2). The typical bands for type I collagen, namely the amide III, I, and A bands appeared at about 1240–1280, 1660 and 3300 cm^−1^, respectively. The amide III band was found in all three collagens, suggesting that they are all dermal collagens [22]. The peak intensity ratios of 1240 cm^−1^ to 1445 cm^−1^ (assigned to amino acid side chains) [23] of DDW- and commercial collagen were similar but higher than that of DAC-collagen. The amide I region indicates unordered random coils [24], which were evident as peaks at 1647 and 1649 cm^−1^ in DAC- and commercial collagen, respectively; this peak appeared at 1649 cm^−1^ in DDW-collagen but was less intense than in the other two collagens. The absorption peaks representing the α-helix structure of collagen also appeared in the amide I band as peaks at 1657 cm^−1^ in DDW- and commercial collagen and at 1659 cm^−1^ in DAC-collagen [25]. The amide A bands (stretching vibration of NH group) [13] of DAC- and commercial collagen appeared at 3327 cm^−1^ and that of DDW-collagen appeared at 3312 cm^−1^.

#### 3.1.3. Diffraction Patterns of Collagens

**Figure 3:**
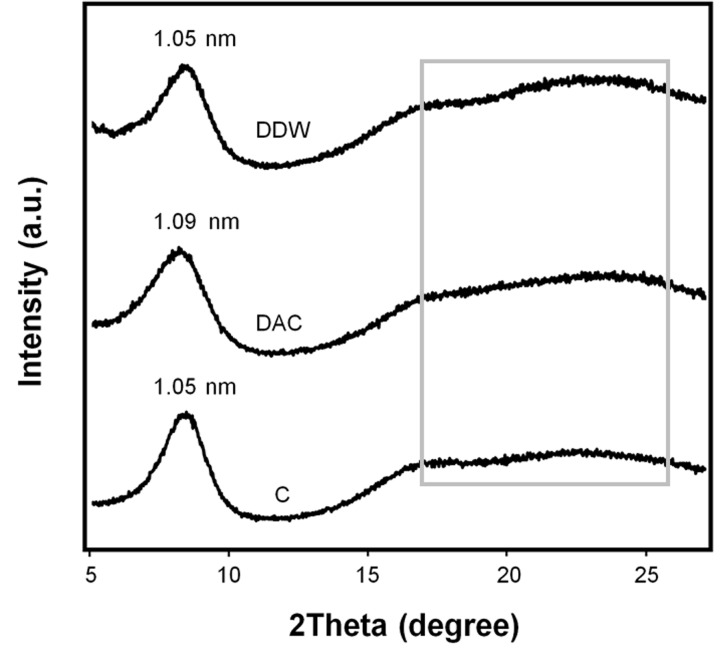
X-ray diffraction patterns of collagens. The sharp peaks represent the distance between the molecular chains of collagen and the broad peaks (in box) represent the amorphous scattering of collagen.

The diffraction patterns of the collagens represented in Figure 3 coincides with the diffraction of native skin collagen [26,27]. The sharp peaks appearing at around 8° indicated that the longest distances between the triple helix molecular chains were 1.09 nm for DAC and 1.05 nm for both DDW- and commercial collagen. However, there were no differences in the broad peaks that appeared around 16–25° (amorphous scatter resulting from unordered components of collagen).

#### 3.1.4. Thermal Characteristics of Collagens

**Figure 4:**
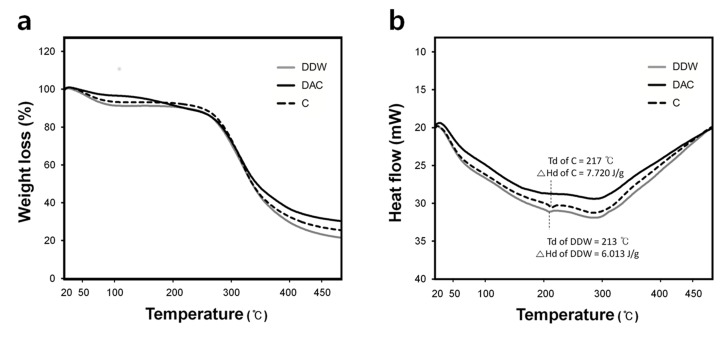
Thermal gravimetric analysis (TGA) and differential scanning calorimetry (DSC) curves of collagens. (**a**) TGA curves indicate the weight loss evolution of collagen with increasing temperature. (**b**) DSC curves represent the heat flow evolution of collagen with increasing temperature.

In the Thermal gravimetric analysis (TGA) thermograms, DAC was represented by a bimodal curve with the highest and lowest weight loss in the 1st and 2nd weight loss regions, respectively, among the studied collagens (Figure 4a, Table 2). The denaturation temperature (Td) and denaturation enthalpy (ΔHd) of DDW and commercial collagen were similar to those previously reported for type I collagen in a dehydrated state (Td = 225 °C, ΔHd = 7.05 J/g) [28]. However, the Td and ΔHd of DAC were not determined (Figure 4b).

### 3.2. Type I Collagen-Based Porous Scaffolds

#### 3.2.1. Porosity and Pore Size of Porous Scaffolds

The porosity of all scaffolds was over 98% (Table 3). The porosity decreased with increasing collagen concentration in the DDW-collagen scaffolds (*p* < 0.0001). In contrast, the porosity of the DAC-collagen scaffolds was lower at 0.5 than at 1% *w*/*v*. Regarding the pore size, the DAC-collagen scaffolds showed a slightly larger average pore size than the DDW-collagen scaffolds at the same concentration, but this was not statistically significant (*p* > 0.05). Both DDW- and DAC-collagen scaffolds exhibited increasing average pore size with an increase in collagen concentration (0.5 vs. 1% *w*/*v* scaffolds, *p* > 0.05; 0.5 and 1% *w*/*v* scaffolds vs. 2% *w*/*v* scaffolds, *p* < 0.001) (Table 3).

#### 3.2.2. Morphologies of Porous Scaffolds

**Figure 5:**
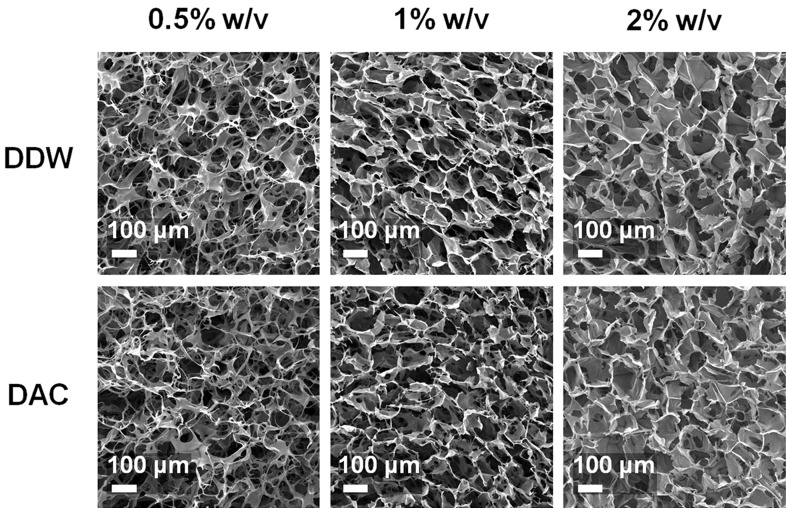
Morphological characteristics of porous scaffolds. Scanning electron micrographs represent the pore morphology of the top surface of the scaffolds.

Despite the partially collapsed walls of the pore structures in all DAC-collagen scaffolds, the pore morphology was more dependent on the collagen concentration than on the dialysis fluid. Indeed, the top surfaces showed remarkably interconnected open pore structures at 0.5% *w*/*v* collagen and uniformly distributed channels with structurally thick pore walls at 2% *w*/*v* concentration (Figure 5).

#### 3.2.3. Physical Characteristics of Porous Scaffolds

For DDW- and DAC-collagen scaffolds, the weight difference increased with an increase in collagen concentration. At 0.5, 1, and 2% *w*/*v* collagen, the DAC-collagen scaffolds were 1.03-, 1.04-, and 1.06-fold lighter than the DDW-collagen scaffolds at the same concentration (0.5% *w*/*v*, *p* > 0.05; 1% *w*/*v*, *p* < 0.01; 2% *w*/*v*, *p* < 0.001). At 0.5% *w*/*v* collagen, the DAC-collagen scaffolds exhibited remarkably lower volume (1.85-fold) and thickness (2.33-fold) than the DDW-collagen scaffolds at the same concentration (*p* < 0.001). At 1% *w*/*v* collagen, the DAC-collagen scaffolds were 1.14-fold smaller and 1.05-fold thinner than the DDW-collagen scaffolds (*p* < 0.001), while at 2% *w*/*v* collagen, the DAC-collagen scaffolds were 1.04-fold larger and 1.04-fold thinner than the DDW- scaffolds (*p* < 0.001) (Table 4).

#### 3.2.4. Cross-Linking of Porous Scaffolds

**Figure 6:**
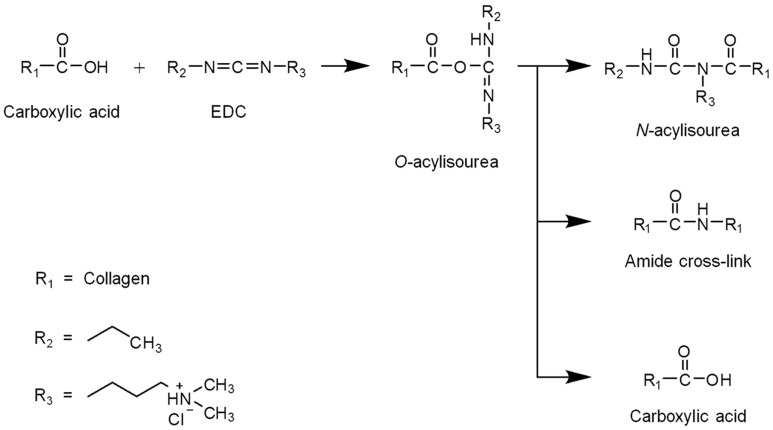
Schematic representation of the reaction between 1-ethyl-3-(3-dimethylaminopropyl) carbodiimide (EDC) and collagens.

**Figure 7:**
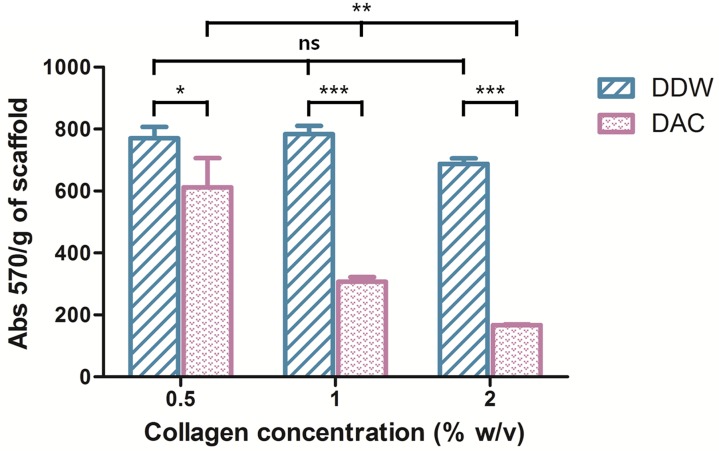
Free amine index of porous scaffolds. *** *p* < 0.001, ** *p* < 0.01, * *p* < 0.05, ns *p* > 0.05.

EDC was used as a protein cross-linking reagent by activating the carboxyl group and forming an amide with the amine groups of collagen (Figure 6) [29]. The remaining free amines were used to determine the degree of cross-linking. A high free amine index indicates a low degree of cross-linking [20]. The free amine indices of the DAC scaffolds were lower than those of the DDW-collagen scaffolds and decreased with increasing collagen concentration (*p* < 0.01). In contrast, there was no notable trend in the indices of the DDW-collagen scaffolds. At 2% *w*/*v*, the DDW-collagen scaffolds showed the lowest free amine index, whereas at 0.5 and 1% *w*/*v*, the free amine indices were comparable (*p* > 0.05) (Figure 7).

#### 3.2.5. Water Absorbance Ability of Porous Scaffolds

**Figure 8:**
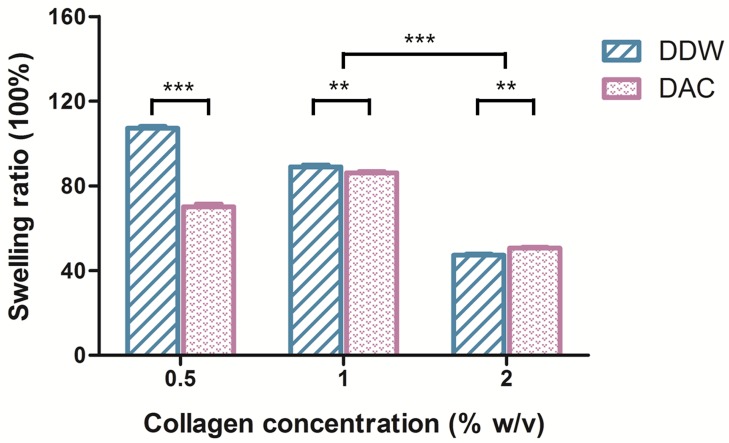
Swelling ratios of porous scaffolds. The weight change of scaffolds after water absorption was measured. *** *p* < 0.001, ** *p* < 0.01.

The DDW-collagen scaffolds exhibited a 1.53-fold higher swelling ratio than the DAC-collagen scaffolds (107.15 ± 1.01 and 70.07 ± 1.29 g_water_/g_dry scaffold_ for DDW- and DAC-collagen scaffolds, respectively, *p* < 0.001) at 0.5% *w*/*v* collagen and a 1.03-fold higher swelling ratio at 1% *w*/*v* collagen (88.96 ± 0.88 and 86.18 ± 0.64 g_water_/g_dry scaffold_ for DDW- and DAC-collagen scaffolds, respectively, *p* < 0.01). In contrast, at 2% *w*/*v* collagen, the DDW-collagen scaffolds exhibited a 1.07-fold lower swelling ratio than the DAC-collagen scaffolds (47.33 ± 0.45 and 50.62 ± 0.44 g_water_/g_dry scaffold_ for DDW- and DAC-collagen scaffolds, respectively, *p* < 0.01) (Figure 8).

#### 3.2.6. In Vitro Degradation of Porous Scaffolds

**Figure 9:**
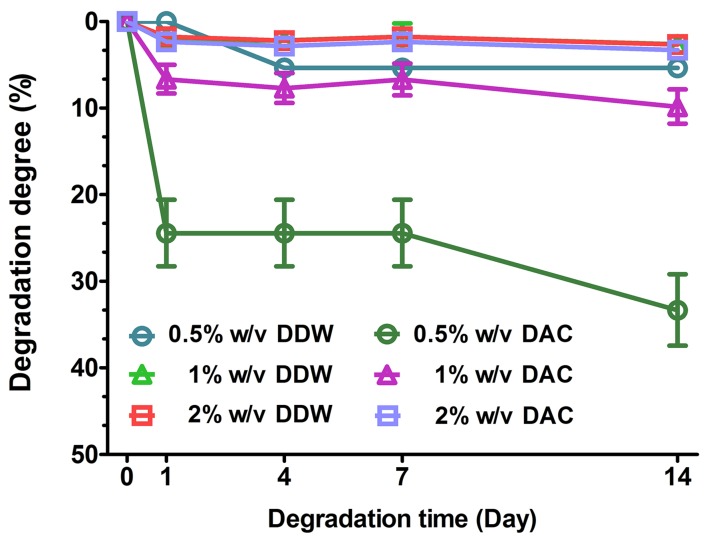
Degradation degree of porous scaffolds. The scaffolds were incubated in 1× PBS with orbital shaking for 14 d and the weight loss of the scaffolds was then determined.

As seen in Figure 9, the DDW-collagen scaffolds exhibited less weight loss than the DAC-collagen scaffolds. Weight loss in the DDW-collagen scaffolds occurred during days 0–1 (1% and 2% *w*/*v*) and days 1–4 (0.5% *w*/*v*), with no marked weight loss after 4 days. However, at 0.5 and 1% *w*/*v*, the DAC-collagen scaffolds showed noticeable weight loss during days 0–1 and 7–14. The differences in weight loss between DDW- and DAC-collagen decreased as the collagen concentration increased at day 14 (0.5% *w*/*v*, *p* < 0.001; 1% *w*/*v*, *p* < 0.01; 2% *w*/*v*, *p* > 0.05).

### 3.3. Cell Culture Studies Using Porous Scaffolds

**Figure 10:**
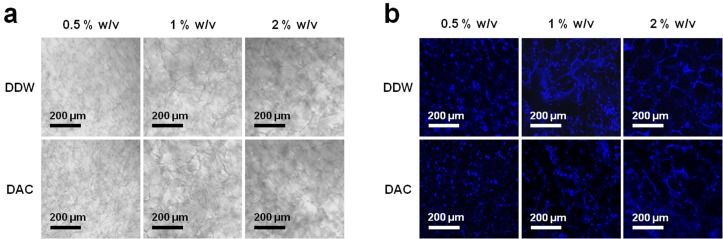
Optical and fluorescence microscope images of adipose-derived stem cells (ADSCs)-seeded porous scaffolds. (**a**) Both DDW- and DAC-collagen scaffolds show a pore structure. (**b**) ADSCs distributed along the pore wall can be observed via DAPI staining (at day 1 after cell seeding).

**Figure 11:**
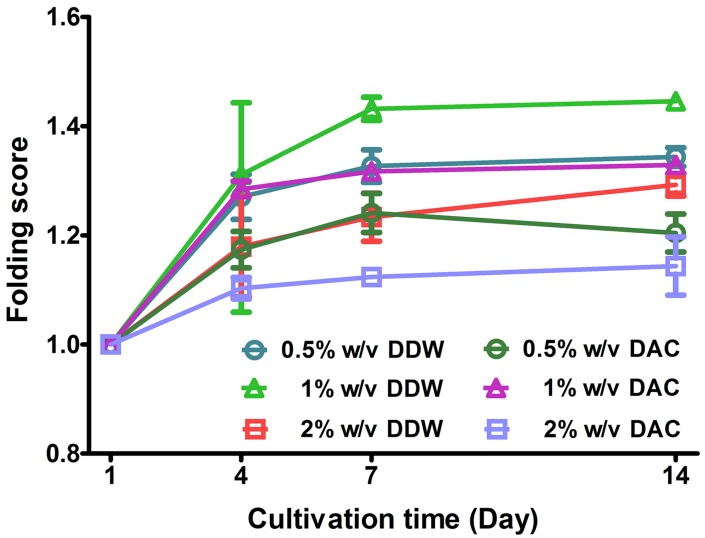
Proliferation of adipose-derived stem cells on porous scaffolds. The folding score at day 1, 4, 7 and 14 was normalized against the cells at day 1.

**Figure 12:**
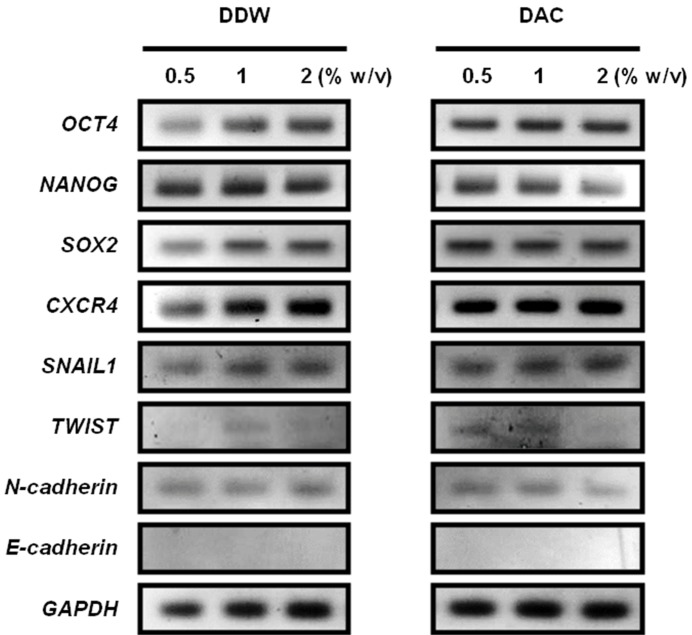
Characterization of adipose-derived stem cells on porous scaffolds by reverse transcriptase polymerase chain reaction analysis. Expressions of pluripotency markers (*OCT4*, *NANOG* and *SOX2*), mesenchymal stem cell marker (*CXCR4*) and epithelial-mesenchymal transition markers (*SNAIL*, *TWIST*, *N-cadherin*, and *E-cadherin*) were determined at day 10 after seeding.

ADSCs cultured on porous scaffolds were distributed along the linings of the pore walls, and there were no evident differences between DDW- and DAC-collagen scaffolds or among the three collagen concentrations (Figure 10). Both DDW- and DAC-collagen scaffolds showed the highest proliferation at 1% *w*/*v* collagen and the lowest proliferation at 2% *w*/*v*, while only the 0.5% *w*/*v* DAC-collagen scaffolds exhibited decreasing proliferation during days 7–14 (Figure 11). At day 10 after cell seeding, the ADSC-seeded scaffolds expressed the pluripotency markers *OCT4*, *NANOG*, and *SOX2* [30] and the MSC marker *CXCR4* [31]. There were no considerable differences in the expression levels of these genes between DDW- and DAC-collagen scaffolds or among the three collagen concentrations. Conversely, the expression of epithelial-mesenchymal transition (EMT) markers varied; *SNAIL* was expressed similarly in the two scaffolds, but *TWIST* was variably expressed, *N-cadherin* was weakly expressed, and *E-cadherin* was not expressed (Figure 12) [32,33,34].

## 4. Discussion

While acetic acid (0.5 M, pH 2–3) can be used exclusively to isolate collagen, only partial hydration of the collagen is achieved, and intermolecular cross-linked collagen fibrils are not cleaved [10,14]. In contrast, pepsin digests the collagen and cleaves the telopeptide region without disrupting its triple-helical structure; in this region, β and γ chains are converted to α1 and α2 chains [8,12]. The protein pattern results indicated that both DDW- and DAC-collagen were type I collagens having α1, α2, β, and γ chains, and they did not suggest any marked influence of acid residue on the relative proportions of the chains. Conversely, its influence on the structural and conformational characteristics of the isolated collagens was significant. In both DDW- and DAC-collagen, α-helices (at ~1660 cm^−1^) were the most intense component of the amide I band. Yet, the unordered structures represented by the peak at 1647 cm^−1^ and the low 1240 cm^−1^/1445 cm^−1^ ratio suggested the denaturation of DAC-collagen [13,24]. In addition, the amide A band appeared at a higher wavelength (3327 cm^−1^) for DAC- than for DDW-collagen (3312 cm^−1^), indicating the weakening of hydrogen bonds in the triple-helical structure of DAC-collagen [35]. Likewise, the longest distance between the triple helix molecular chains of DAC-collagen, shown in the diffraction pattern, indicated the denaturation of collagen by acetic acid. Collectively, these results show that removing acetic acid residue from isolated collagen can lead to a highly ordered structure with strong hydrogen bonding [13].

Similar to the structural stability results, the thermal stability of DAC-collagen was lower than that of DDW-collagen; acetic acid residue attributed to an additional weight loss for DAC-collagen (in the 1st weight loss region) and resulted in DAC-collagen exhibiting the lowest proportion of pure collagen (in the 2nd weight loss region). In the DSC thermograms, a weak denaturation enthalpy in DAC-collagen indicated that acid residue induced strong thermal disruption of collagen and leaded to the formation of random chain of gelatin [28].

Porous scaffolds that resist enzymatic degradation can be fabricated using three broad steps, namely, freezing, lyophilizing, and cross-linking [18,36]. The porous structure of the scaffold is designed in the freezing process, where increasing the solute amount decreases the porosity and pore size [37,38]. After the cross-linking process, the porosity and pore size of the initial product are reduced, and this is correlated with shrinkage of the scaffold [39]. All scaffolds created in this study were highly porous (>98% porosity). The average pore size of all DAC-collagen scaffolds was larger than that of the DDW-collagen scaffolds, which may be attributed to the presence of acetic acid residue, as acid has been shown to induce repulsion between collagen molecules [40]. Furthermore, acetic acid increased the ice crystal size on the freezing process, resulting in a further increase in pore size with weak pore walls [38]. Notably, in this study, the pore size was increased with increasing collagen concentrations in both groups. Considering the relationship between pore size and scaffold shrinkage, as mentioned above, the decrease of pore size with a decrease in collagen concentration is highly correlated with the decrease in scaffold volume after cross-linking.

EDC optimally reacts with carboxyl groups under slightly acidic conditions (pH ~ 5.5) [29,41,42]. In this context, the low free amine indices of the DAC-collagen scaffolds compared to those of the DDW-collagen scaffolds reflect the low pH. However, decreasing the free amine indices in DAC-collagen scaffolds may not indicate an increase in amide bonds (i.e., cross-linking) between two collagens [29]. The higher probability of O-acylisourea in the DAC-collagen than in the DDW-collagen scaffolds may increase the probability of its rearrangement into N-acylisourea, and acetic acid may participate in the reaction, decreasing the cross-linking between collagen molecules. Thus, the likelihood of increasing the strength of DAC-collagen scaffolds via more extensive cross-linking [43] may be low.

At all concentrations, the DDW-collagen scaffolds were heavier and thicker than the DAC-collagen scaffolds, and the volume of the DDW scaffolds (about 200–350 μL) was either larger than or similar to that of the DAC scaffolds. These physical characteristics of DDW-collagen scaffolds are associated with their high structural stability. As observed from the swelling degree of the 0.5% *w*/*v* scaffolds, a highly flexible structure due to a low collagen concentration is believed to facilitate the expansion of the scaffold network structure. Consequently, the scaffold can hold a large amount of water (DDW-collagen scaffold). However, despite a high flexibility, because of low structural stability, the scaffold cannot retain the absorbed water (DAC-collagen scaffold). Conversely, at a high collagen concentration (2% *w*/*v*), the porous network structure showed low expansion, and therefore its water absorbance ability decreased. Particularly, the lowest swelling behavior, which was evident in the 2% *w*/*v* DDW-collagen scaffolds, indicated they had a relatively rigid structure and, accordingly, a high structural stability.

In the degradation behavior of scaffolds, there were no significant differences among the scaffolds of the DDW group (at day 14), In contrast, weight loss increased with decreasing collagen concentrations in the DAC group (at day 14). In particular, the weight loss of 0.5% *w*/*v* DAC-collagen scaffolds was 6.22-fold higher than that of 0.5% *w*/*v* DDW-collagen scaffolds, indicating that the scaffolds with a highly flexible structure but low structural stability had low resistance to mechanical stimulation.

The high porosity and pore interconnectivity of both DDW- and DAC-collagen scaffolds reflected their amenity to cell infiltration and cell proliferation [44,45,46]. ADSCs cultured on porous scaffolds were well distributed inside their pore wall regardless of collagen concentration or dialysis medium. In relation to cell proliferation, both DDW- and DAC-collagen scaffolds showed the highest cell proliferation at a 1% *w*/*v* collagen concentration and the lowest proliferation at 2% *w*/*v*. The combined effect of high volume, high swelling capacity, and low degradation behavior (1% *w*/*v* scaffolds) may induce proliferative potential, whereas markedly reduced swelling capacity (2% *w*/*v* scaffolds) may reduce this potential. The 0.5% *w*/*v* DAC-collagen scaffolds displayed a decrease in cell proliferation from day 7 that may be linked to the weight loss that occurred when degradation restarted.

The RT-PCR data suggest that the ADSCs maintained the characteristics of MSCs in all scaffolds for 10 d; there was no marked influence of dialysis medium or collagen concentration on the expression of MSC or pluripotency markers, and although the EMT makers were variably expressed, these results did not suggest transdifferentiation of the ADSCs. However, the low volume, thickness, and swelling capacity and high degradation behavior of the DAC-collagen scaffolds may be critical for stable cell behaviors. Thus, DDW-based collagen scaffolds may be more suitable for supporting the long-term culture of cells.

## 5. Conclusions

In this study, we found that the structural and thermal characteristics of the isolated collagens were dependent on the dialysis medium and that acetic acid induced collagen denaturation. At the same concentration, DDW- and DAC-collagen scaffolds had comparable pore morphologies and were highly porous. However, the acetic acid associated with DAC promoted scaffolds with larger pore sizes and lower free amine indices compared to those of DDW-collagen scaffolds. The physical characteristics of the scaffolds indicated a higher structural stability for DDW-collagen than for DAC-collagen scaffolds. DDW-collagen scaffolds exhibited the ability to absorb and retain large amounts of water and had high resistance to weight loss by mechanical stimulation. Both DDW- and DAC-collagen scaffolds showed the potential to maintain MSC characteristics of ADSCs, but the proliferative activity of ADSCs was higher in the DDW-collagen scaffolds. These results suggest that the elimination of acetic acid residue from isolated collagen is recommended to produce collagen scaffolds that provide a stable environment for cells and cell therapy-related applications.

## Figures and Tables

**Table 1:** Primer sequences for RT-PCR analysis of adipose-derived stem cells.

Gene Name	Primer Sequence	Accession No.	Amplicon Size (bp)	Annealing Temperature (°C)	Cycle
*OCT4* ^1^	S 5′-cgt gaa gct gga gaa gga gaa gct g-3′	AF268617	245	60	34
	A 5′-caa ggg ccg cag ctc aca cat gtt c-3′				
*NANOG*	S 5′-caa agg caa aca acc cac tt-3′	NM_024865	394	60	34
	A 5′-att gtt cca ggt ctg gtt gc-3′				
*SOX2* ^2^	S 5′-aac ccc aag atg cac aac tc-3′	NM_003106	100	60	34
	A 5′-gct tag cct cgt cga tga ac-3′				
*CXCR4* ^3^	S 5′-ggt ggt cta tgt tgg cgt ct-3′	BC020968.2	324	60	34
	A 5′-tcg atg ctg atc cca atg ta-3′				
*SNAIL1*	S 5′-ttt acc ttc cag cag ccc ta-3′	BC012910	415	54	34
	A 5′-cca ggc tga ggt att cct tg-3′				
*TWIST*	S 5′-agt ccg cag tct tac gag ga-3′	NM_000474.3	222	54	34
	A 5′-cat ctt gga gtc cag ctc gt-3′				
*N-cadherin*	S 5′-gac aat gcc cct caa gtg tt-3′	NM_001792.3	354	54	34
	A 5′-acc cac aat cct gtc cac at-3′				
*E-cadherin*	S 5′-tgg aca ggg agg att ttg ag-3′	NM_004360.3	458	60	34
	A 5′-agg ctg tgc ctt cct aca ga-3′				
*GAPDH* ^4^	S 5′-tcc atg aca act ttg gta tc-3′	NM_002046	452	55	34
	A 5′-tgt agc caa att cgt tgt ta-3′				

^1^*OCT4* octamer-binding transcription factor 4; ^2^ *SOX2* SRY (sex determining region Y)-box 2; ^3^ *CXCR4* Chemokine (C-X-C motif) receptor 4; ^4^ *GAPDH* glyceraldehyde 3-phosphate dehydrogenase.

**Table 2:** Thermal gravimetric data for collagens.

Sample	T_1st_ (°C) ^1^	Weight Loss 1st (%) ^2^	Weight Loss 2nd (%) ^3^
DDW	173	8.8	67.5
DAC	243	11.4	56.6
C	159	6.8	65.8

^1^ The temperature after losing water and volatile compounds; ^2^ Weight loss at temperatures ranging between 20 °C and T_1st_; ^3^ Weight loss at temperatures ranging between T_1st_ and 450 °C.

**Table 3:** Porosity and pore size of scaffolds.

Sample (% *w*/*v*)	Porosity (%)		Pore Size (μm)	
	DDW	DAC	DDW	DAC
0.5	99.20 ± 0.01	98.58 ± 0.02	74.63 ± 9.05	77.04 ± 11.54
1	99.01 ± 0.01	98.91 ± 0.01	79.05 ± 12.02	81.80 ± 12.24
2	98.14 ± 0.01	98.31 ± 0.01	91.03 ± 20.49	97.65 ± 17.12

**Table 4:** Weight, thickness and volume of porous scaffolds.

Sample (% *w*/*v*)	Weight (mg)		Thickness (mm)		Volume (μL)	
	DDW	DAC	DDW	DAC	DDW	DAC
0.5	1.93 ± 0.06	1.87 ± 0.06	1.77 ± 0.03	0.76 ± 0.02	200.46 ± 3.90	108.34 ± 2.10
1	3.87 ± 0.06	3.73 ± 0.06	2.42 ± 0.03	2.30 ± 0.01	321.53 ± 4.50	282.09 ± 1.70
2	7.77 ± 0.06	7.30 ± 0.00	2.60 ± 0.02	2.49 ± 0.01	344.53 ± 2.38	356.60 ± 1.10
